# Perceived Stress in Adults Aged 65 to 90: Relations to Facets of Time Perspective and *COMT* Val^158^Met Polymorphism

**DOI:** 10.3389/fpsyg.2018.00378

**Published:** 2018-03-22

**Authors:** Michael Rönnlund, Elisabeth Åström, Rolf Adolfsson, Maria G. Carelli

**Affiliations:** ^1^Department of Psychology, Umeå University, Umeå, Sweden; ^2^Department of Clinical Science, Division of Psychiatry, Umeå University, Umeå, Sweden

**Keywords:** perceived stress, time perspective, Catechol-O-Methyltransferase, older adults, Val^158^Met polymorphsim

## Abstract

This study examined the relation between perceived stress and time perspective (views of past, present, future) in a population-based sample of older adults (65–90 years, *N* = 340). The Perceived Questionnaire (PSQ index) was used to measure stress and the Swedish version of the Zimbardo Time Perspective Inventory (S-ZTPI) was used to operationalize time perspective. Unlike the original inventory, S-ZTPI separates positive and negative aspects of a future time perspective and we hypothesized that the Future Negative (FN) scale would be important to account for variations in stress. Additionally, associations with Catechol-O-methyltransferase (*COMT*) Val^158^Met polymorphism were examined, motivated by prior associations of this single nucleotide polymorphism (SNP) with stress (or “anxiety”) related personality traits. In line with the hypotheses, FN was the strongest predictor of PSQ index scores in multiple regression analyses. In a related vein, the dichotomization of the unitary Future scale increased the association between PSQ scores and a measure of deviations from a balanced time perspective, i.e., the difference between a proposed optimal and observed ZTPI profile. Finally, higher levels of stress as well as higher scores on FN were observed in *COMT* Val/Val carriers, at least among men. This suggests a shared dopaminergic genetic influence on these variables. Collectively, the results demonstrate that perceived stress is closely linked to time perspective and highlight the need to take negative aspects of a future temporal orientation into account to understand this relation.

## Introduction

Physiological and hormonal reactions in response to external or internal “stressors” that threaten homeostasis, may be adaptive in the short run, but durable stress may exert an adverse influence on many aspects of mental and physical health (Schneiderman et al., 2005). High levels of perceived stress tend to be less prevalent among older compared with younger or middle-aged adults (e.g., Bergdahl and Bergdahl, 2002; Vasunilashorn et al., 2005), however, still could have serious consequences. For example, elevated and durable stress in old age may accelerate memory decline, in particular when combined with a genetic vulnerability (e.g., Peavy et al., 2007), increase the risk of conversion from Mild Cognitive Impairment (MCI) to dementia (Peavy et al., 2013) and predict mortality (Aldwin et al., 2011). Although there are links between stress exposure and disease outcomes, these associations are not easily deconstructed (cf. O’Donovan et al., 2013). Hence, it becomes increasingly important to identify factors that modulate stress in late adulthood.

Acute as well as chronic stress relate to a variety of environmental factors and aspects of a person’s life situation. At the same time, levels of perceived stress show considerable stability over decades (Lindgren et al., 2016) are moderately heritable (Federenko et al., 2006) and related to dispositional factors (e.g., Ebstrup et al., 2011; Lee, 2012) that are under substantial genetic influence (e.g., Jang et al., 1996). Given recent evidence that perceived stress and aspects of personality indeed share a considerable amount of genetic influences (Luo et al., 2017) a search for common genetic associations are of interest. In the present study we focused on perceived stress in older adults in relation to an individual differences factor regarded to be stable and trait-like, namely time perspective, and the relations of both variables to a single nucleotide polymorphism (SNP) of the *COMT* gene.

### Time Perspective

Human experiences may be considered to be organized through a cognitive framework that involves three temporal frames: past, present, and future. According to Lewin (1951) these time frames allow us to make sense of past experiences and anticipate future outcomes, thereby influencing how we think, feel, and behave in the present.

In the theoretical framework by Zimbardo and Boyd (1999), time perspective refers to the relative focus on and valence assigned to each of the three time frames. To capture individual differences in time perspective, they developed the Zimbardo Time Perspective Inventory (ZTPI). This inventory involves 56-items and comprises five subscales: (1) Past Positive (PP), that reflects a positive and nostalgic view of the personal past, (2) Past Negative (PN), which reflects a negative or aversive view of the past, (3) Present Hedonistic (PH), which involves pleasure seeking without particular consideration of future consequences, (4) Present Fatalistic (PF), which captures a helpless attitude toward the present, and an external locus of control, and (5) Future, which reflects a general future orientation that involves optimism and a strive for future goals and rewards.

A growing number of studies, so far largely restricted to young adults, demonstrate that time perspective as operationalized by ZTPI is linked to a variety of adaptive/maladaptive behaviors and aspects of mental health. For example, a predominant present focus was related to risky driving (Zimbardo et al., 1997) and more frequent use of alcohol and tobacco (Keogh et al., 1999). By contrast, a predominant future orientation was linked to positive health behaviors, including medication adherence (Sansbury et al., 2014) and reduced likelihood of early-onset substance use (Wills et al., 2001). Higher scores on PN was, on the other hand, a prominent predictor of depressive symptoms (e.g., Desmyter and De Raedt, 2012) and of depressive disorders (Oyandel and Buela-Casal, 2014). Thus, several maladaptive behaviors and forms of mental ill-health may relate to the fact that the individual is “stuck” in some specific temporal frame or attitude.

In addition to scores on the individual ZTPI dimensions, recent studies often report values for a measure of deviations from a balanced time perspective (DBTP; Stolarski et al., 2011). This is to provide an estimate of the degree to which an individual’s constellation of ZTPI scores across all five subscales show a global time perspective bias. More specifically, DBTP is computed as a total sum of deviations from a so called balanced, time perspective (BTP), a profile characterized by high scores on PP, moderately high scores on PH and Future and low scores on the negatively valenced scales (Zimbardo and Boyd, 1999, 2008; Stolarski et al., 2011). Thus, even minor biases evident across several of the ZTPI scales might add up to a significant DBTP.

### Time Perspective and Stress

By now, the association between time perspective and health behaviors has been well documented, and attention has turned to understand time as mediator of social/contextual effects on behavior and to understand the mechanism by which time perspective influences health behavior (Joireman et al., 2012). It is then possible that time perspective has an influence on disease-mediating states such as stress. So far, a limited number of studies examined the relation between time perspective as conceptualized by ZTPI and stress.

In a first study (Papastamatelou et al., 2015), a Greek version of the ZTPI was administered to a group of students, together with the Perceived Stress Scale (Cohen et al., 1983). Correlational analyses revealed a substantial association (*r* = 0.50) between scores on the stress scale and DBTP suggesting that higher levels of stress are associated with an aggregate time perspective bias. As judged from bivariate associations, higher scores on PN and PF were the primary factors behind this pattern, and in multiple regression analyses both scales remained significant predictors of stress, with the highest β-value (0.49) for PN. The same basic pattern was evident when groups high or low in level of perceived stress were compared.

The second study (Olivera-Figueroa et al., 2015) examined cortisol levels in relation to the separate ZTPI dimensions and in relation to DBTP. Measurement of cortisol levels was made before and after exposure to the Trier Social Stress Test. A significant negative relationship (*r* = -30) between a measure reflecting total HPA-axis systemic output (AUCg; Area under the curve with respect to ground; see Pruessner et al., 2003) and DBTP was observed, with no association with a measure of reactivity to the stress-induction (AUCi; area under the curve with respect to increase). Usually acute stress is associated with elevated cortisol levels, but the negative association between DBTP and AUCg indicates that those with higher DBTP scores had lowered cortisol levels. Atypically low levels of cortisol, i.e., hypocortisolism, is regarded to reflect chronic stress exposure, and observed in conditions such as chronic fatigue syndrome, PTSD (Edwards et al., 2011), and bipolar disorder (Maripuu et al., 2014). Olivera-Figueroa argued that high DBTP may be indicative of hypocortisolism and chronic stress. Of the separate ZTPI scales, only PN was significantly (negatively) correlated with AUCg. Thus, the study indicated that DBTP is associated with a biological marker of stress, and that high scores on PN (cf. Papastamatelou et al., 2015), may be the most characteristic temporal bias in individuals with (chronic) stress.

Finally, two studies examined time perspective in relation to previous or recent stressful life events. In Holman (2015), significantly higher scores on PN and PH were observed for individuals who had experienced a stressful event earlier in life compared with individuals who had not experienced such an event. Similarly, participants who had experienced a more recent stressful event also scored higher on PN. The validity of these results were limited by use of retrospective reports; recall of events as well as time perspective may, for example, be influenced by current mood state. More informative in this regard, a longitudinal study initiated after the 9/11 terrorist attack (Holman et al., 2016) repeatedly administered a few items drawn from the past, present and future ZTPI scales up to 3 years after the collective trauma. In essence, the results indicated high stability of inter-individual differences of time perspective scores, supporting the dispositional character of time perspective, but also dynamic changes in PN in those who reported higher levels of ongoing stress. Interestingly, this held true for younger, but not for older, adults, consistent with other observations (e.g., Isaacowitz et al., 2006; Charles et al., 2009) that older adults are less influenced by stressful situations and negative stimuli than younger adults.

Taken together, using quite different means to conceptualize and measure stress, available studies indicate a significant association between stress and facets of time perspective. Stress was associated with DBTP in two studies, and with elevated scores on PN in particular. The findings by Holman et al. (2016) highlight that although facets of time perspective may be regarded as dispositional factors that influence perception of stress, adverse events (traumas) could also influence aspects of time perspective (e.g., PN), particularly in younger adults.

### Distinguishing Future Positive and Future Negative

Notably, none of the first two studies observed an association between stress and scores on the ZTPI Future scale. The single Future scale of the original inventory mainly reflects a positive and constructive orientation toward the future, though, and as noted by E. Paul Torrance, future oriented thinking may influence emotional state depending on valence:

“Positive images of the future are a powerful and magnetic force... They draw us on and energize us, give us courage and will to take on important initiatives. Negative images of the future also have a magnetism. They pull the spirit downward in the path of despair...” (Torrance, 1983, p 72).

Along similar lines of reasoning, and in analogy with a subdivision of the past and present time frames into scales with positive and negative valence (i.e., PP vs. PN), Carelli et al. (2011) extended the Swedish version of the instrument (S-ZTPI) so as to differentiate positive and negative aspects of a future temporal perspective. Essentially, the original Future scale was kept (except for two items) and referred to as Future Positive (FP). Eight items were added for a new Future Negative (FN) scale and the two items that were dropped from the Future scale were in addition included. The FN scale aims to capture a broadly aversive view of the future (“To think about my future makes me sad.”; “The future contains too many boring decisions that I do not want to think about”) including negative expectations with regard to attainment of future goals (“I do not know how I will be able to fulfill my goals in life”) and worry (“If things don’t get done on time, I don’t worry about it”; reverse coded item) as specific aspects of a negative FTP.

With regard to construct validity, confirmatory factor analyses indicated that the new subscale was factorially distinct from the other S-ZTPI subscales, including FP (Carelli et al., 2011). Initial evidence of criterion validity included a strong negative association with a dependent decision making style (i.e., searching for advice and guidance from others; Scott and Bruce, 1995) that was unrelated to FP. Conversely scores on a scale capturing a rational decision making style scale (searching for information and logically evaluating alternative decision-making options) exhibited a positive correlation with FP (and Future) (Carelli et al., 2011) unlike FN. Additionally, scores on FN were more strongly related to maladaptive coping strategies such as substance use and denial in adolescents than FP scores (Blomgren et al., 2016) and exhibited a strong negative association with a latent well-being factor that was unrelated to FP (Rönnlund et al., 2017).

Of main concern in the present study, we hypothesized that a negative FTP may be particularly important to account for the variations in stress. For example, in transactional models (Lazarus and Folkman, 1984) stress is thought of as resulting from an imbalance between perceived demands and coping resources. The individual will experience stress when the demands exceed her or his (perceived) ability to cope with stressors at hand. Thus, stress may often be related to anticipatory negative thinking to the extent that the demands (e.g., some to-be-performed tasks or deadline to meet), are ahead in time. In general, therefore, individuals with a propensity toward future fears or worries (i.e., higher FN) should be more prone to experience symptoms of stress.

Of interest at this point, higher scores on FN, in addition to PN (cf. Papastamatelou et al., 2015), was observed in individuals with high (>8) versus low (<8) scores on the Beck Anxiety Inventory (Åström et al., 2014). This is in line with the expectation that anxiety, in which worry and uncertainty in regard to the future may be considered characteristic, and which partly overlaps with stress conceptually, should exhibit elevated scores on the FN. Relatedly, O’Connor and colleagues found that perceived stress was associated with generating more negative future expectations (O’Connor et al., 2004) as measured by the Future Thinking Task (MacLeod et al., 1997). This finding further underlines that negative views of the future are an important dimension of FTP in relation to perceived stress.

### The Present Study

The aim of this study was to examine relations between perceived stress and time perspective using the extended version of the ZTPI (S-ZTPI) in a sample of adults aged 65 or older. As noted by Osmanovic-Thunström et al. (2015) this age range is still scarcely studied with regards to perceived stress, a notable omission given aging of the populations. Based on previous studies of young adults we expected stress to be associated with DBTP. Based on the study by Papastamatelou et al. (2015), we furthermore expected scores on PN to be positively correlated with a measure of perceived stress. Critically, we hypothesized that a more aversive attitude toward the future (FN) not considered in prior studies, would exhibit a unique positive relationship with stress. In a related vein, we expected that the subdivision of the original ZTPI Future scale into positive and negative subscales would increase the associations between a measure of DBTP and stress.

To elucidate the relation between stress and time perspective we additionally examined both factors in relation to the functional Catechol-O-Methyltransferase (*COMT*) polymorphism (Val^158^Met). This was motivated by previous indications of an association with anxiety (or stress) related personality traits (Lee and Prescott, 2014). The polymorphism is characterized by a substitution of methionine (Met) in place of Valine (Val) which results in a decrease in the *COMT* enzyme (Lachman et al., 1996). In turn, the slower breakdown of catecholamines in Met carriers should result in higher levels of extracellular levels of dopamine in the prefrontal cortex (Weinberger et al., 2001). This matter presumably accounts for findings that Met-carriers (or Met homozygotes) tended to outperform Val homozygotes on measures of executive functioning (e.g., de Frias et al., 2005; Barnett et al., 2007) and episodic recall (e.g., de Frias et al., 2004).

It was originally hypothesized that the advantage of Met (i.e., with regard to executive functioning) might be paired with a disadvantage in regard to processing of negative stimuli and be coupled with “anxiety-related traits” (e.g., Neuroticism; Stein et al., 2006). An increasing number of results contradict the latter hypothesis, though. A meta-analysis by Lee and Prescott (2014) indicated heterogeneity in the outcome across studies, but found that, at least in male samples, the Met allele (Met/Met) was associated with lower levels of Neuroticism (Caucasian samples) and Harm Avoidance (Asian samples). Thus, even though results regarding the relation between *COMT* and personality factors linked to stress are mixed and may be modulated by factors such as sex and ethnicity, the Met allele may actually have a protective role in regard to stress-related dispositions, at least in men. In this case, Val homozygotes may be expected to exhibit higher levels of perceived stress and score higher on the facets of time perspective we hypothesize to be linked to elevated stress, i.e., PN and FN.

## Materials and Methods

### Participants

The data were collected as part of the sixth wave in the Betula Prospective Cohort Study (Nilsson et al., 1997, 2004), when a version of the (S-ZTPI) was added to the battery. The participants in this study were initially recruited via random selection from the population registry in Umeå municipality (for details regarding initial screening criteria, sampling, and measurements, see Nilsson et al., 1997).

A total of 340 participants from sample 1 and 3, aged 65, 70, 75, 80, 85, or 90 years at date of test, 184 women (54.1%) and 156 men (45.9%), met the present inclusion criteria: (1) complete or near-complete data on the measure of stress (Perceived Stress Questionnaire) and time perspective, (2) Mini-Mental State Examination (MMSE, Folstein et al., 1975) score ≥ 24, and (3) being genotyped for the *COMT* Val^158^Met polymorphism The allelic distribution in the sample was: Met/Met (*n* = 108), Met/Val (*n* = 156), Val/Val (*n* = 76), genotype frequencies that did not deviate from Hardy-Weinberg Equilibrium (HWE), χ^2^(2) = 1.87, *p* = 0.17. In the main analyses, we merged the first two groups into one (i.e., Met carriers).

### Measures

#### Perceived Stress Questionnaire (PSQ)

The Swedish version of the Perceived Stress Questionnaire (PSQ; Levenstein et al., 1993; Bergdahl and Bergdahl, 2002) was used to measure stress. The PSQ consists of 30 items. Each item involves a description of a stress-related experience (“you feel tense”; “you feel that problems pile up”) that is rated with regard to its frequency of occurrence. A four-point scale is used: *never* (coded as 1), *almost never* (2): *sometimes* (3): *often*, and *usually* (4). Two alternative versions (or instructions) are used, involving the frequency of occurrence during the last month (“PSQ-recent”) or during the last year (“PSQ-general”). In the present study the “PSQ-recent” was used. Research has identified various subfactors (e.g., Levenstein et al., 1993) but as demonstrated by Rönnlund et al. (2015) the putative first-order factors are reflective of a strong first-order (i.e., general stress) factor that motivates the use of a general index of stress (PSQ index). High internal consistency for the total scale (Cronbach’s α = 0.90) has been reported (Bergdahl and Bergdahl, 2002; Rönnlund et al., 2015). The PSQ-index (Levenstein et al., 1993) was used as a measure of global stress and is computed as: (PSQ sum of raw scores-30)/90. The resulting score ranges from 0 (lowest level of perceived stress) to 1 (highest level of perceived stress). Cut-off values for levels of stress established on the basis of large scale Swedish sample (Bergdahl and Bergdahl, 2002) are: <0.34 (low stress), 0.34–0.46 (moderate stress) and >0.46 (high stress).

#### Swedish Zimbardo Time Perspective Inventory (S-ZTPI)

Swedish Zimbardo Time Perspective Inventory (S-ZTPI) (Carelli et al., 2011) consists of 64 items. Each item involves a statement that reflects one of six time dimensions: PP (e.g., “Familiar childhood sights, sounds, smells often bring back a flood of wonderful memories”), PN (e.g., “Painful past experiences keep being replayed in my mind”), PH (e.g., “I believe that getting together with one’s friends to party is one of life’s important pleasure”, PF (e.g., “Fate determines much in my life”); FP (e.g., When I want to achieve something, I set goals and consider specific means for reaching those goals”), and FN (e.g., “To think about my future makes me sad”). For each of the statements, the participant is requested to rate how characteristic it is of his/her own view on five-point Likert scale, ranging from *very uncharacteristic* (coded 1) to *very characteristic* (coded as 5). Confirmatory factor analyses of the six factor version and validity evidence included differential associations of the scales with decision making styles in predicted directions (Carelli et al., 2011). Internal consistencies ranged from 0.65 (PF) to 0.94 (PN) across subscales (Carelli et al., 2011).

Deviations from a Balanced Time Perspective (DBTP) based on the original ZTPI was computed according to the formula (Stolarski et al., 2011):

$$\begin{array}{c} \sqrt{{\left(\mathrm{oPN}-\mathrm{ePN}\right)}^{2}+\;{\left(\mathrm{oPP}-\mathrm{ePP}\right)}^{2}+\;{\left(\mathrm{oPF}-\mathrm{ePF}\right)}^{2}+\;{\left(\mathrm{oPH}-\mathrm{ePH}\right)}^{2}+\;{\left(\mathrm{oF}-\mathrm{eF}\right)}^{2}} \end{array}$$

where, in accord with Stolarski et al. (2011), o = optimal (ideal) score and e = empirical (observed) score.

The corresponding measure (DBTP-E) based on the extended version of the ZTPI (S-ZTPI) perspective was computed according to the formula (cf. Rönnlund et al., 2017):

$$\begin{array}{c} \sqrt{{\left(\mathrm{oPN}-\mathrm{ePN}\right)}^{2}+\;{\left(\mathrm{oPP}-\mathrm{ePP}\right)}^{2}+\;{\left(\mathrm{oPF}-\mathrm{ePF}\right)}^{2}+\;{\left(\mathrm{oPH}-\mathrm{ePH}\right)}^{2}+\;{\left(\mathrm{oFP}-\mathrm{eFP}\right)}^{2}+\;{\left(\mathrm{oFN}-\mathrm{eFN}\right)}^{2}} \end{array}$$

Optimal scores (o) were in line with prior studies (Stolarski et al., 2011; Rönnlund et al., 2017) set to: oPN = 1.95, oPP = 4.6, oPF = 1.5, oPH = 3.9, oF/oFP = 4.0, and oFN = 1.8. The ZTPI o-values were scores corresponding to specific percentile ranks in a cross-cultural database (see Stolarski et al., 2011). The value for FN was defined by the same percentile (10th) adopted for PN and PF in the former study (Rönnlund et al., 2017).

#### DNA

Genomic DNA was isolated from whole blood by use of Qiagen Genomic DNA Purification Kit (Qiagen Inc., Chatsworth, CA, United States). Polymerase chain reactions were carried out using HotstarTaq polymerase (Qiagen). This was done in a total volume of 20 l1 containing 1.5 mM MgCl2, 0.15 lM primers (fw: 50 -TCA CCA TCG AGA TCA ACC CC-30, rev: 50 -ACA ACG GGT CAG GCA TGC A-30), and approximately 50 ng genomic DNA. Following a 15 min denaturation step at 95°C, 45 cycles were performed including 30 s at 94°C, 30 s at 62°C, and 30 s at 72°C. PCR products were genotyped with a Pyrosequencer PSQ 96 and the PSQ 96 SNP Reagent Kit (Pyrosequencing, Uppsala, Sweden; Nordfors et al., 2002), by use of the sequence primer 50 -TGG TGG ATT TCG CTG-3.

## Results

### Bivariate Association and Descriptive Statistics of the Study Variables

Zero-order correlations of the variables in the study are presented in **Table 1** together with descriptive statistics.

**Table 1:** Zero-order correlations and descriptive statistics (*M, SD*) of the variables in the study.

Variable	*M*	*SD*	1	2	3	4	5	6	7	8	9	10	11	12
(1) PSQ index	0.17	0.12	1											
(2) Past Positive	3.60	0.51	–0.24^***^	1										
(3) Past Negative	2.29	0.60	0.54^***^	–0.21^***^	1									
(4) Present Hedonistic	2.91	0.44	0.04	0.27^***^	0.19^**^	1								
(5) Present Fatalistic	2.53	0.53	0.30^***^	0.06	0.40^***^	0.36^***^	1							
(6) Future Positive	3.25	0.44	0.09	0.19^**^	0.19^***^	0.02	–0.07	1						
(7) Future Negative	2.51	0.56	0.58^***^	–0.03	0.65^***^	0.17^**^	0.44^***^	0.34^***^	1					
(8) DBTP	2.17	0.51	0.36^***^	–0.59^***^	0.48^***^	–0.28^***^	0.47^***^	–0.14^*^	0.30^***^	1				
(9) DBTP-E	2.34	0.57	0.48^***^	–0.55^***^	0.57^***^	–0.18^***^	0.55^***^	–0.22^***^	0.48^***^	0.90^**^	1			
(10) Sex (*f* = 1, *m* = 0)	–	–	0.10	0.01	0.02	0.03	0.14^*^	–0.04	0.11^*^	0.03	0.08	1		
(11) Age	72.71	6.33	0.07	0.06	0.16^**^	–0.01	0.26^***^	0.01	0.16^**^	0.14^**^	0.19^***^	0.03	1	
(12) COMT (Val/Val = 1)^a^	–	–	0.11^*^	–0.06	0.10	0.01	–0.03	–0.01	0.11^*^	0.01	0.05	0.00	0.03	1

*^∗^*p* < 0.05, ^∗∗^*p* < 0.01, ^∗∗∗^*p* < 0.001. f = female, m = male, ^*a*^Met = 0.*

Notably, PSQ-index was negatively associated with scores on PP (*r* = -0.24, *p* < 0.001), but positively associated with PN (*r* = 0.54, *p* < 0.001) as well as PF (*r* = 0.30, *p* < 0.001). Critically, Future Positive was unrelated to PSQ index (*r* = 0.09; the original Future scale, based on two additional items, yielded a similar value of *r* = 0.06). By contrast, FN showed a strong association with PSQ index (*r* = 0.58, *p* < 0.001). Similarly, the measure of deviation from a balanced time perspective based on a six factor version of the ZTPI with dichotomized future scales (DBTP-E) showed a stronger association with PSQ-index (*r* = 0.48, *p* < 0.001) than the measure based on the five original ZTPI scales (DBTP, *r* = 0.36, *p* < 0.001; *z* = 5.03, *p* < 0.001 for the difference in *r-*value).

**FIGURE 1:**
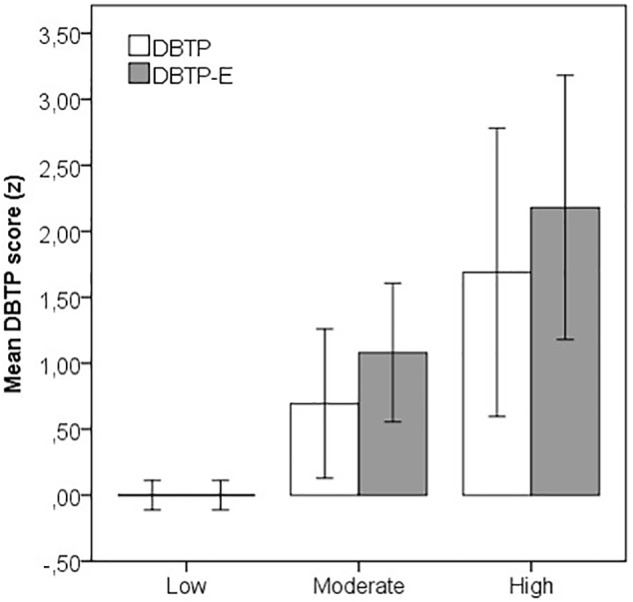
Mean (*z*) scores for two measures of deviations from a balanced time perspective without (DBTP) or with (DBTP-E) dichotomized Future scales across grouping of stress (low, moderate, high) based on cutoff values for the PSQ index (Bergdahl and Bergdahl, 2002).

As an alternative means to illustrate the relationship between the two versions of the DBTP measure, groups based on the cutoff values for PSQ index (Bergdahl and Bergdahl, 2002) were in addition compared. For this purpose, scores on each measure were transformed to *z* scores on basis of *M/SD* of the participants categorized as low in stress level (PSQ index < 0.34; *n* = 309). The resulting mean *z* scores with 95% confidence intervals for participants categorized as low (PSQ index < 0.34, moderate (PSQ index 0.34–0.46, *n* = 23) or high (PSQ index > 0.46, *n* = 8) in stress level are shown in **Figure 1**.

Clearly, means for both measures of DBTP differed substantially across level of stress, with larger differences for DBTP-E compared to DBTP (*z* > 2 for the low-high stress contrast). Notably, DBTP-E, but not DBTP, differed significantly for participants high versus moderate in stress levels [*t*(29) = -1.87, *p* = 0.035, one-tailed test; *p* = 0.074 for DBTP] in spite of the small *n*s.

Returning to the values in **Table 1**, the results moreover indicate that the PSQ index was significantly associated with variations in *COMT* (*r* = 0.11, *p* = 0.037) with higher scores in Val homozygotes (*M* = 0.19, *SD* = 0.11) compared with Met carriers (*M* = 0.16, *SD* = 0.12). Val homozygotes additionally scored higher on FN (*M* = 2.63, *SD* = 0.55) than Met carriers (*M* = 2.48, *SD* = 0.56, *r* = 0.11, *p* = 0.047). Once more a comparison based on cutoff values for the PSQ index was deemed to be of interest. The moderate and high stress groups (*n* = 31) were collapsed for this purpose. Of the Val homozygotes, 15.8% of were classified as being moderate/high in stress level compared with 7.5% of the Met carriers, χ^2^(1) = 5.26, *p* = 0.02.

### Multiple Regression Analyses of PSQ Index

To estimate the relative and total contribution of all included variables (demographic factors, *COMT* polymorphism, and time perspective scales) we performed hierarchic regression analyses with PSQ index as the regressor. The demographic variables (age, sex) were entered in a first step (Model 1). Next (Model 2), *COMT* (Val homozygote = 1, Met carrier = 0) was entered. Finally, the S-ZTPI scales were entered together as a block (Model 3). Variance Inflation Factors (VIFs; all values < 2.3) and values for tolerance (all values > 0.45) were not indicative of concerns in regard to multicollinearity (cf. Menard, 1995). The results of the regression analyses are summarized in **Table 2**.

**Table 2:** Summary of regression analyses of PSQ index.

	Model 1			Model 2			Model 3		

Variable	β	*t*	*p*	β	*t*	*p*	β	*t*	*p*
Age	0.070	1.29	0.197	0.073	1.35		–0.037	–0.85	0.396
Sex (female = 1)	0.093	1.71	0.087	0.093	1.73	0.085	0.034	0.88	0.429
COMT (Val/Val = 1)				0.115^∗^	2.14	0.033	0.032	0.73	0.447
Past Positive							–0.149^*^	–3.15	0.002
Past Negative							0.225^*^	3.97	0.000
Present Hedonistic							–0.054	–1.15	0.253
Present Fatalistic							0.039	0.72	0.470
Future Positive							–0.054	–1.32	0.253
Future Negative							0.446^*^	7.18	0.000
Total *R*^2^				0.027			0.425		
Δ*R*^2^	0.014			0.013			0.398		
*p*-value (F_change_)	0.095			0.033			0.000		

*^∗^p < 0.05.*

**FIGURE 2:**
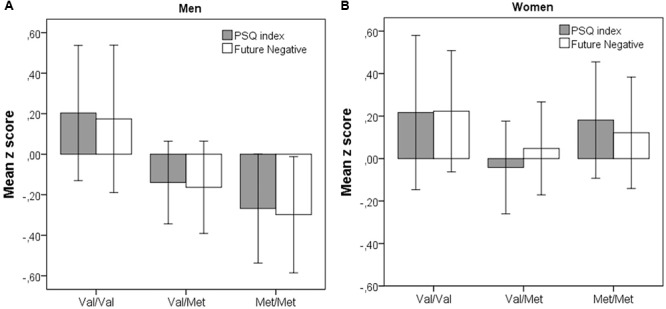
Mean PSQ index and Future Negative (*z* scores) as a function of COMT group (Val/Val, Val/Met, Met/Met) for men **(A)** and women **(B)**. Error bars represent 95% confidence interval.

None of the demographic variables (Model 1) were significantly associated with PSQ index. However, being Val homozygote (Model 2), was a significant predictor of higher stress over and beyond the demographic factors (β = 0.115, *p* = 0.033). Given previous indications of sexually dimorphic relation of *COMT* with personality factors, preliminary analyses included an interaction term. Whereas it was non-significant, stratified analyses (controlling for age in the first step) revealed a significant association of *COMT* with PSQ index in the slightly smaller sample of men (β = 0.179, *p* = 0.025), but not in women (β = 0.068, *p* = 0.36), with a similar pattern for FN, i.e., a significant association with *COMT* in men (β = 0.164, *p* = 0.038) but not in women (β = 0.067, *p* = 0.36). Additionally, as illustrated in **Figure 2**, only in men (A) was a clear dose-response relationship observed, increased Met load (coded as 0, 1, 2) being related to lower PSQ scores (β = 0.19, *p* = 0.018, adjusted for age) and lower FN scores (β = 0.18, *p* = 0.021, adjusted for age).

In the third and final step (Model 3), addition of S-ZTPI subscales resulted in substantial increment in *R*^2^, of about 40%. Of the six subscales, the significant unique predictors in the final model were PP, that was associated with lower PSQ score, PN, and FN, with the strongest unique association (β-value = 0.44) for FN. Finally, it might be noted that *COMT* showed no association with PSQ index (*p* = 0.47) following entry of the time perspective dimensions in Model 3. The latter is consistent with a shared association of *COMT* genotype and with time perspective, FN in particular, in prediction of PSQ score. Consistent with the latter interpretation, entry of FN alone in step/Model 3 removed the association between *COMT* and PSQ index.

## Discussion

The objective of this study was to examine the relation between perceived stress and facets of time perspective in a sample of older adults. The results agree with those obtained in prior studies in several respects. First, levels of stress were substantially associated with aggregate measures of DBTP, suggesting that the basic pattern observed in younger adults (Olivera-Figueroa et al., 2015; Papastamatelou et al., 2015) generalizes to older adults. Second, in bivariate as well as multivariate analyses higher scores on PN were associated with elevated stress. This is consistent with prior studies involving various indicators of stress (Olivera-Figueroa et al., 2015; Papastamatelou et al., 2015) and in agreement with a broader set of findings designating high PN as a characteristic across a range of mental health problems and disorders (van Beek et al., 2011) for which stress may be regarded as a precipitating factor, including depression (e.g., Desmyter and De Raedt, 2012) and bipolar disorder (Oyandel and Buela-Casal, 2014). In the present study a unique (negative) association between stress and PP was additionally observed, indicating that access to positive aspects of the personal past might serve to buffer against stress. In a related vein, nostalgia, that may be regarded as a specific aspect of a PP time perspective, has been designated as a resource that may serve to counter negative emotions evoked by stressors, for example by increasing self-esteem, feelings of social connectedness, and perceptions of meaning in life (for a review, see Routledge et al., 2013). Importantly, the present results add to the literature by demonstrating a critical role of a FTP in stress. Whereas a general future orientation (Future or FP) was unrelated to stress, as in previous studies (Olivera-Figueroa et al., 2015; Papastamatelou et al., 2015), scores on FN turned out to be the strongest predictor of stress among the individual S-ZTPI subscales. In a related vein, a measure of DBTP based on separate future scales was more closely associated with stress (cf. Rönnlund et al., 2017 for subjective well-being) compared with a measure based on a single Future dimension (Stolarski et al., 2011). Additionally, the extended DBTP measure (DBTP-E) more clearly differentiated between cutoff-based groups (i.e., low, moderate or high in stress). The present indications of need to differentiate a unitary FTP possibly relate to the observation by Kozik et al. (2015) that the FTP scale (Carstensen and Lang, 1996; Unpublished) which assesses the individual’s future time extension, the extent to which the future is “open”, may be separated into “opportunities” vs. “limitations” of which low focus on the latter was selectively associated with a reduced level of hair cortisol in a sample of older adults.

Another key finding, further supporting the relation of FN to stress, was that of a common association of the variables with *COMT* Val^158^Met polymorphism. More specifically, Val homozygotes exhibited a more aversive view of the future and reported higher levels of stress than Met carriers. To our knowledge, this is the first study to report a genetic association of aspects of time perspective as assessed by the ZTPI/S-ZTPI. There was some indication of the genetic association to be more apparent in men, for which a dose-response function, with successively lower stress levels and FN scores with increased *COMT* Met load (i.e., lowest in Met/Met carriers) was observed. As such, the results appear consistent with meta-analytic evidence of higher Neuroticism and Harm Avoidance in Val compared with Met homozygotes in males (Lee and Prescott, 2014). Based on prior associations with Neuroticism and Harm Avoidance, it is not fully clear whether the present associations between *COMT* and FN is related temporal processes or negative affectivity in general. No significant association with PF and PN, which should reflect general negativity, was observed though. It might also be noted that anticipatory worry is a facet of Harm Avoidance.

Certainly, the link of *COMT* genotype to stress and FN needs to be replicated in other studies. Provided that it is substantial, it may imply a common link to prefrontal functions. Green et al. (2013) for example, indicated neural mediation of the link of COMT genotype to cognitive control and IQ. Executive control, or inhibition factor, may in turn be critical to regulate negative future thinking, for example to inhibit worry (Beckwé et al., 2014), a specific form of future-oriented negative thought process, thereby minimizing perceived stress. Indeed, a recent study (Zajenkowski et al., 2016) found that an executive control factor reflecting two inhibition measures was inversely related to DBTP score. Future studies are required to examine the proposed links between *COMT*, cognitive control, a negative future orientation, and stress.

## Limitations

Even though strengths of the present study, including a population-based sample and a comprehensive measurement of the FTP, might deserve to be highlighted, it clearly has limitations. In accordance with the theoretical framework underlying ZTPI (Zimbardo and Boyd, 1999) we assume that preexisting time perspective biases are a vulnerability factor of stress. However, it is important to acknowledge that the data are cross-sectional. This precludes any firm conclusions regarding directionality of the influences. Results by Holman et al. (2016) may be taken to suggest that the reversed causal influence, i.e., from adverse events/stress to time perspective is more limited in older adults, though. To estimate the relative strength of potential bidirectional influences, longitudinal studies involving repeated measurements of the constructs are required. Ideally, such a study would include multiple indicators of stress, e.g., measures of cortisol as a complement to a measure of perceived stress. Finally, even though the sample was population-based, we screened for cognitive dysfunction and individuals with severe health problems may have been less likely to turn up at the assessments. Potentially, such factors may have masked an age-related increment in levels of perceived stress that may be expected in the unselect population of older persons, due to increasing health-related concerns (Osmanovic-Thunström et al., 2015).

## Conclusion

The present study demonstrated that perceived stress is substantially linked to an individual’s time perspective organization. The results add to prior observations that time perspective is an important correlate of well-being and facets of mental health among older adults (e.g., Shmotkin and Eyal, 2003; Desmyter and De Raedt, 2012; Webster et al., 2014). In particular, several aspects of our results, including the finding of a common genetic association (*COMT*) of perceived stress and score on FN, highlight a need to take a negative aspects of a FTP into account to understand the relation between time perspective and stress. Given these patterns, stress is also an important candidate as a disease-mediating state in relations between time perspective and health-related outcomes. Future studies may be able to pinpoint the link between stress and aspects of time perspective, including common neural substrates. Finally, even though the present study points to a role of genetic factors in stress and time perspective, the results indicate a differential vulnerability to stress, not that levels of stress are fixed. Hence, interventions designed to alleviate stress are of interest. A link between (dispositional) mindfulness, which is a well-established correlate of stress and amenable to training (e.g., Khoury et al., 2015), and DBTP (Stolarski et al., 2016; see also Droit-Volet and Heros, 2017) is interesting in this regard.

## Ethics Statement

This study was approved by the regional ethic review board, Umeå, with written informed consent from all subjects. All subjects gave written informed consent in accordance with the Declaration of Helsinki.

## Author Contributions

MR performed the data analyses and wrote a first draft. EÅ, RA, and MC made critical revisions of the manuscript.

## Conflict of Interest Statement

The authors declare that the research was conducted in the absence of any commercial or financial relationships that could be construed as a potential conflict of interest.
